# Application of Intraoperative Optical Coherence Tomography Technology in Anterior Segment Surgery

**DOI:** 10.1155/2022/1568406

**Published:** 2022-04-08

**Authors:** Sang Beom Han, Yu-Chi Liu, Karim Mohamed-Noriega, Jodhbir S. Mehta

**Affiliations:** ^1^Department of Ophthalmology, Kangwon National University School of Medicine, Kangwon National University Hospital, Chuncheon, Republic of Korea; ^2^Singapore National Eye Centre, Singapore; ^3^Singapore Eye Research Institute, Singapore; ^4^Department of Ophthalmology, Yong Loo Lin School of Medicine, National University of Singapore, Singapore; ^5^Department of Ophthalmology, University Hospital, Faculty of Medicine, Autonomous University of Nuevo Leon, Monterrey, Mexico

## Abstract

The use of optical coherence tomography (OCT) technology in anterior segment diseases allows for precise assessment of the changes following anterior segment surgery. Advances in microscope-integrated OCT systems have allowed the utilization of intraoperative OCT (iOCT) in anterior segment surgeries, i.e., cornea, cataract, and refractive surgery. iOCT has enabled real-time precise visualization of anterior segment tissues as well as interactions between surgical instruments and ocular tissue; thus, the device can facilitate surgical procedures and provide valuable information for decision-making during anterior segment surgeries. In this review, the authors will introduce studies regarding the development of iOCT technology and its application in various anterior segment surgeries. Multiple studies have shown the efficacy of the iOCT for intraoperative assistance and guidance, suggesting the potential of the device for optimizing the surgical outcomes after cornea, cataract, and refractive surgery.

## 1. Introduction

To date, evaluation of ocular tissues and tissue-instrument interactions during anterior segment surgery has depended on directing visualization using an operating microscope. Although technological development enables coaxial operating microscopes to show high-resolution images, they cannot provide detailed three-dimensional (3D) visualization of the anterior segment structures. [1, 2] Hence, operating microscopes have limited ability to provide depth information for anterior segment tissues. [1–3] Although intraoperative slit beam examination can be helpful for depth evaluation, it does not have a high enough resolution for precise measurement of tissue depth. [1] In addition, tissues behind the cloudy media, such as opaque cornea, cannot be visualized using traditional operating microscopes, which may be problematic in cases with corneal edema or haze. [1–4].

The application of optical coherence tomography (OCT) technology, which can provide high-resolution cross-sectional images of anterior segment structures, has allowed precise assessment of anterior segment tissues; this may be advantageous for the diagnosis and treatment of various anterior segment diseases. [2, 3, 5] Introduction of the intraoperative OCT (iOCT) technology to anterior segment surgery is expected to allow real-time precise visualization of anterior segment tissues and tissue-instrument interactions, which may contribute to improved anatomical and visual outcomes. [2, 3, 5–7] In particular, iOCT can be advantageous in cases with corneal opacity in which structures underneath the cornea are difficult to visualize with an operating microscope. [2, 3, 5, 7] Detailed intraoperative visualizations of these tissues can be helpful for more precise surgical procedures and improved visual outcomes in various anterior segment surgeries, such as corneal surgeries including penetrating keratoplasty (PK), deep anterior lamellar keratoplasty (DALK), Descemet stripping automated endothelial keratoplasty (DSAEK), and Descemet membrane endothelial keratoplasty (DMEK), cataract, and refractive surgery. [1–3, 5, 7].

In this review, we provide an overview of the advances in iOCT systems and the application of these devices in various anterior segment surgeries. The potential future developments and applications of iOCT are also discussed.

## 2. Development of Intraoperative OCT Technology

Several developments have been made to overcome the barriers to the intraoperative use of OCT. [2, 7, 8] First, conventional OCT modalities are stationary table top systems and lack portability; thus, integration into the ophthalmic surgical microscope is difficult. [2]To address this problem, intraoperative imaging using lightweight handheld OCT was first introduced. [9] However, handheld OCT can never provide “real-time” intraoperative imaging because surgeons must cease surgical procedures when obtaining OCT images or request an assistant to take the images. [7, 9] Limited stability of the handheld device and involuntary hand movement can be associated with difficulty in image acquisition and decreased image quality. [2] Introduction of a nonsterile handheld device to a sterile surgical field can also increase the risk of infection. [2, 5].

**Figure 1:**
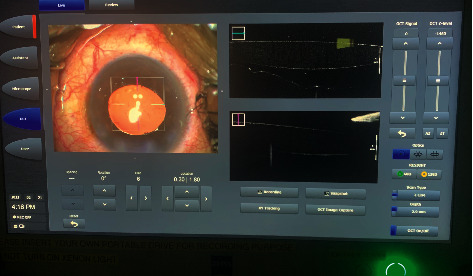
Display of an image of the intraoperative optical coherence tomography (iOCT) integrated into the microscope during the cataract surgery.

To address this problem, researchers introduced a method of mounting a lightweight handheld OCT probe to the operating microscope, which can enable improved stability, higher image quality, faster image acquisition, and enhanced sterility. [10, 11] However, the mounted OCT system needs extra space for the machine in the operating room and an additional foot switch to control the OCT probe. [2, 5] Moreover, in the microscope-mounted OCT system, surgeons still need to discontinue the procedures before image capture.10. To allow “real-time” intraoperative image acquisition without surgical delay, microscope-integrated OCT (MIOCT), in which OCT probes are integrated into the microscopic optics, have been developed (Figure 1). [1, 2] In MIOCT systems, surgeons can control the pattern of OCT image scan, such as the region of interest, orientation, and size, with a microscope foot-pedal in which the OCT system is also integrated. [12, 13].

To date, three systems of MIOCT are commercially available: Rescan® 700 (Cirrus OCT system built on Lumera 700 microscope; Carl Zeiss Meditec, Oberkochen, Germany), [12, 14] OPMedT® (OPMedT OCT system, Haag-Streit Hi-R 1000G-microscope, Haag-Streit Surgical GmbH, Wedel, Germany) [15], and EnFocus® (Bioptigen, Leica, Wetzlar, Germany). [1, 2].

## 3. Application of Intraoperative OCT in Anterior Segment Surgery

Several studies have shown the efficacy of iOCT in various anterior segment surgeries [1, 2, 5, 7, 8, 10, 13–37]. Researchers indicated that information obtained using iOCT was helpful for intraoperative decision-making and selection of surgical procedures [1, 14, 15, 19, 38].

In a large prospective study including 275 eyes, Ehlers et al. [10] revealed that a portable OCT system mounted on a microscope can be helpful for visualization of anterior segment structures during anterior segment surgery, particularly in lamellar keratoplasty procedures [10]. In a subsequent study, they demonstrated that the MIOCT enabled real-time visualization of anterior segment tissues and tissue-instrument interaction [12, 14].

### 3.1. iOCT in Corneal Transplantation

In PK, iOCT can enable the visualization of structures beneath the hazy and irregular cornea that are difficult to identify with an operating microscope [7]. During PK, iOCT can allow real-time display of the host-graft interface, which can be helpful for appropriate apposition at the interface.8. Eguchi et al. [39] showed that iOCT can be useful for the detection of iridocorneal adhesion and iris incarceration that can occur during PK [39]. iOCT can also provide information regarding the location of the suture needle penetrating the corneal layers, which can lead to a secure suture by adjusting the needle depth [7].

**Figure 2:**
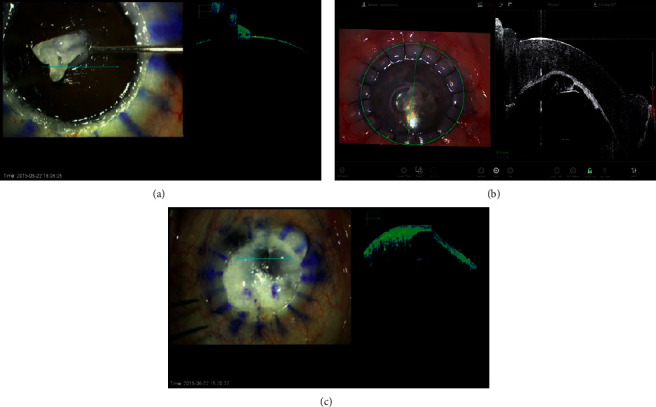
Application of the iOCT in DALK surgery. (a) Visualization of the residual stromal bed. (b) Confirmation of the attachment between the graft cornea and recipient bed (c) visualization of the tissue underneath the cloudy cornea using the iOCT during the viscodissection.

In DALK, iOCT can display all surgical steps and facilitate intraoperative decision-making [10, 12, 14, 20, 35, 40]. During the DALK procedure, iOCT can provide real-time cross-sectional visualization of deep needle injection, intrastromal bubbles, deep dissection plane, residual stromal bed, Descemet's membrane (DM), and interface fluid (Figure 2) [7, 10, 12, 14, 17, 20, 26, 35, 40]. Scorcia et al. [31] demonstrated that the iOCT enabled detailed evaluation of the dissection depth of the corneal stroma, which may improve the success rate for big-bubble formation in DALK [31]. In cases in which big bubble was difficult to attain, e.g., cases with corneal scars involving DM, iOCT enables precise assessment of the depth and uniformity of the stromal dissection plane, which is critical for the achievement of a residual bed with appropriate thickness and smooth surface. [1] In DALK cases, in which viscodissection is performed for stromal dissection, iOCT may be particularly helpful as it can visualize the tissues underneath the cloudy cornea caused by viscoelastic bubbles [20]. De Benitos et al. [20] also showed that iOCT enabled the achievement of big-bubble and completely detached DM in a case with irregular corneal thinning and scarring (Figure 2(c)). [20] They also reported that iOCT enabled visualization of intrastromal fluid retention that was invisible with the operating microscope in a case with diffuse corneal scarring due to chemical injury [20]. Eguchi et al. [39] demonstrated that iOCT can also detect iris protrusion caused by misdirected air into the posterior chamber at the end of DALK that can lead to acute intraocular pressure elevation due to angle closure [39].

**Figure 3:**
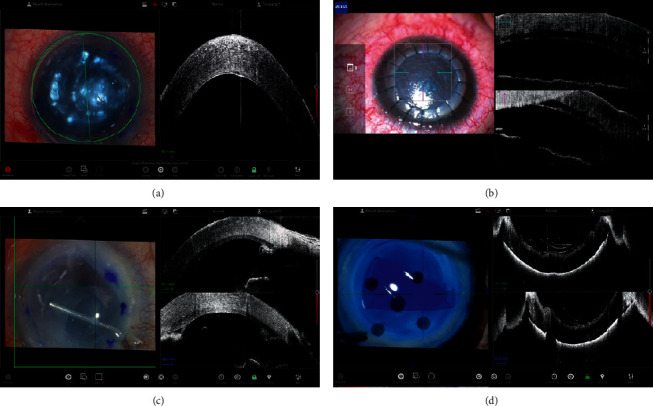
Application of the iOCT in DSAEK and DMEK surgery. (a) Confirmation of the attachment of the endothelial graft during DMEK. (b) Visualization of the detached endothelial graft cornea in DMEK. (c) Visualization of the peripheral anterior synechiae during DSAEK. (d) Visualization of the donor fold before donor graft insertion in DMEK.

In DSAEK and DMEK, iOCT can provide critical information from intraoperative decision-making in all surgical steps [7, 12, 14, 19, 29, 41, 42], which may lead to enhanced anatomical and visual outcomes. [10, 12–15, 19, 38] In both DSAEK and DMEK, iOCT can visualize donor-recipient attachment and residual interface fluid, which may help achieve tight apposition and prevent graft dislocation (Figure 3) [1, 7, 10, 14]. Shazly et al. [32] reported that iOCT may be advantageous for confirming the attachment of the endothelial graft to the host cornea, particularly in cases with severe corneal opacity. Studies have revealed discordance between surgeon's impressions using the operating microscope and information obtained using iOCT in 18–50% of cases, suggesting a potentially critical role of iOCT in endothelial keratoplasty procedures [10, 14]. A prospective study revealed that information provided by iOCT regarding graft malposition or presence of interface fluid led to the modification of intraoperative decision-making or additional surgical maneuvers, such as corneal sweeping, anterior chamber re-bubbling, graft reposition, or additional peeling of residual host DM in 43% of DSAEK or DMEK cases [14].

Juthani et al. [43] demonstrated that transient residual interface fluid quantified using iOCT during DSAEK had correlation with textural interface opacity postoperatively [43]. Moreover, the amount of residual interface fluid measured using iOCT was associated with an increased rate of early graft nonadherence, indicating the potentially critical role of iOCT for immediate detection and removal of interface fluid [44, 45]. iOCT can also be helpful for confirmation of central placement of endothelial grafts and detection of abnormal tissue particles between the graft and host cornea, which may result in an increased success rate and decreased rejection risk [1, 7]. iOCT can be advantageous in cases with abnormal anterior segment structures after multiple ocular surgeries, such as corneal irregularity and opacity, peripheral anterior synechiae, and vitreous incarceration, particularly for determining the relationship between the endothelial graft and the iris or vitreous [7].

In DMEK, iOCT can be a valuable tool for verification of graft orientation, particularly in cases with severe corneal edema or haze [5, 7, 14, 15, 19, 38]. Based on the scrolling configuration of the endothelial graft visualized using iOCT, the DMEK orientation can be easily confirmed prior to the identification of the orienting marker (Figure 3(d) [14, 15, 19, 38, 41, 46]. Thus, with the use of iOCT, the marking of the donor tissue may become unnecessary, which can be beneficial for the preservation of donor corneal endothelial cells [1, 14, 15, 19, 38, 41]. iOCT can also help shorten the learning curve for DMEK [12].

### 3.2. Corneal Trauma and Other Corneal Surgeries

iOCT can be helpful in emergency surgery for corneal trauma. In cases of corneal laceration, iOCT can facilitate secure corneal sutures by demonstrating the needle depth during suture procedures [7]. The modality can also be useful for assessment of the depth of the foreign body in the cornea, which can facilitate effective removal of the foreign body and reduce the risk of complications, such as damage to the surrounding corneal tissue or displacement of the foreign body to the anterior chamber [7]. The depth of corneal opacity can be precisely estimated using iOCT during emergency operations in cases of chemical or thermal burns.[7].

iOCT can help visualize the cleavage plane between the nodule and underlying corneal tissue in image-guided lamellar keratectomy for the surgical treatment of the Salzmann nodular degeneration [14]. In the Bowman layer transplantation, a new procedure for the treatment of advanced keratoconus, iOCT might enable the visualization of the dissection plane in spite of the air-endothelial reflex being obscured [47]. Siebelmann et al. [48] reported that iOCT enabled assessment of corneal opacity and surface smoothness through real-time imaging during phototherapeutic keratectomy [48].

Intracorneal pathology can also be precisely isolated and located for biopsy using iOCT [49]. Ruland et al. [50] showed that iOCT was helpful for the identification and excision of retrocorneal fibrous membranes following PK. In cases of acute corneal hydrops in keratoconus, iOCT facilitated the micropuncture of intrastromal fluid pockets combined with compression sutures and gas tamponade, which resulted in immediate reattachment of DM [33]. During insertion of Intacs® for keratoconus, iOCT can be used for assessment of the depth of the corneal incision and visualization of the corneal channel for the implant [14]. In the corneal cross-linking for keratoconus, iOCT can be used for the measurement of changes in corneal pachymetry [23, 28].

## 4. Refractive Surgery

In small-incision lenticule extraction (SMILE), iOCT can allow real-time visualization of the desired dissection plane [51]. It can also enable intraoperative identification of the intrastromal lenticule and the distinguishment of the lenticule from the overlying stromal cap and underlying stromal bed, resulting in complete isolation of the lenticule [37]. This can be particularly advantageous in difficult cases, for example, cases with an edematous overlying cap [51]. Intraoperative complications, such as incomplete lenticule separation, cap-lenticule adhesion, lenticule remnants, and creation of false planes, can be easily detected using iOCT, which can lead to immediate corrective procedures and enhanced surgical outcomes [37, 51].

During the implantation of an implantable collamer lens (ICL), iOCT can be used for the visualization and assessment of the vault of the ICL. [1] Titiyal et al. [36] reported that intraoperative vaulting measured with iOCT had a close correlation with postoperative vaulting and can be helpful for the detection of extreme vaulting. [36] It enhances the safety of the surgical procedure by providing a real-time display of intraoperative manipulations. [36].

## 5. Cataract Surgery

Corneal incisions for cataract surgery can be visualized with iOCT. [12] As iOCT can easily identify the location of implants, it can be helpful for verification of correct placement of the intraocular lens (IOL). [10, 14] Nagpal et al. [52] recently revealed that the gap between the IOL and the posterior capsule can be successfully measured using iOCT, which may be helpful for evaluation of the dynamics for the space between the IOL and the posterior capsule.

The device can also provide information regarding calculations of optimal IOL. [12] Hirnschall et al. [53] reported that iOCT enabled more accurate measurement of anterior chamber depth and determination of postoperative lens position, which resulted in better refractive outcomes. In patients with a previous radial keratotomy scar, application of the iOCT during cataract surgery may be helpful for monitoring of the stability of the radial scar and early detection of the dehiscence of the scar, which requires immediate sutures [54, 55].

In cases of posterior polar cataracts, iOCT can be a valuable tool to improve the safety of the surgery by detecting pre-existing posterior capsular dehiscence during cataract surgery and preventing lens drop [56]. In pediatric patients with posterior lens opacities, iOCT can allow the categorization of the morphology of the posterior capsule and the posterior lens cortex, as well as the determination of the integrity of the posterior capsule, which can provide guidance for surgical strategy and increase the safety of the surgery [57]. Similarly, iOCT can be useful for categorization of the white cataract into four types and predicting the intraoperative dynamics according to the type, which can provide valuable information for the selection of surgical strategies for safe surgery, particularly in cases of uneventful completion of capsulorhexis [58].

During IOL scleral fixation, iOCT can enable visualization of the scleral flap preparation and assessment of the preparation depth, which results in enhanced stability of the fixated IOL [25]. iOCT can also be helpful in the adjustment of the IOL tilt during the IOL scleral fixation [22]. Using RESCAN 700, Fukumoto et al. [22] demonstrated that the use of iOCT was associated with the significantly reduced angle of the IOL tilt, which might result in decreased lenticular astigmatism and improved visual outcome. [22].

With the development of robotic surgery for cataracts, iOCT integrated with an intraocular robotic surgical system can help improve the safety and efficacy of automated robotic cataract surgery. [18, 59] The images obtained with iOCT can enable real-time intraoperative monitoring and intervention of robotic surgery by ophthalmic surgeons. [18] With further technological development, iOCT integrated with the robotic surgical system is expected to contribute to the enhanced precision and safety of fully automated robotic cataract surgery. [59].

## 6. Future Developments

Although iOCT must be beneficial for the success of various anterior segment surgeries, the cost has been the biggest hurdle for its widespread use. [1] In addition, all three commercially available iOCT devices are integrated into the operating microscope produced by the same manufacturer. Thus, an additional cost is incurred for the utilization of the devices in the operating room. [1] With the rapid increase in the intraoperative application of iOCT, we expect that technological development and mass production may lower the cost of the device.

During the use of iOCT, light scattering and shadowing caused by metallic surgical instruments can seriously limit visualization of the interaction between ocular tissues and instruments and a detailed view of tissues under the instruments. [2, 12, 60] Thus, the development of novel materials for minimizing the impact of instruments is necessary. [61] Compensation of the disturbing effect of the surgical instruments using software methods, such as image processing or adjustment using artificial intelligence, can be an alternative. [6].

The development of a software platform that enables automated tracking of the region of interest and automated analysis of the captured image is also necessary. [2] Automated tracking can allow surgeons to concentrate on the surgical procedures without the burden of manual aiming for the region of interest. [62] Protocols for automated analysis of captured images may enable precise assessment of the changes in the ocular tissues associated with surgical procedures. [45] For instance, an automated protocol for segmentation and quantification of interface fluid during DSAEK can be used for the prediction of anatomical and visual outcomes. [45].

With advances in technology of corneal tomography, new iOCT systems that can provide more precise images with minimal delays are being developed. Instead of the currently used spectral-domain OCT system, swept-source OCT is expected to provide more detailed images of ocular tissues during surgery with enhanced acquisition speed. [2] In addition, the application of ultrahigh resolution OCT can enable visualization of anterior segment structures at a microscopic level, which may result in more precise surgical procedures and enhanced surgical outcomes. [63].

## 7. Conclusion

The development of iOCT technology may allow real-time precise visualization of ocular tissues and tissue-instrument interactions during anterior segment surgery, which can contribute to improved visual and anatomical outcomes. [2, 8] The device can be significantly advantageous in cases with corneal opacity, particularly during lamellar keratoplasties, such as DALK, DSAEK, and DMEK. [8] Application of the iOCT in cataract and refractive surgery can also enable precise measurement of the ocular parameters, which may lead to optimal visual outcomes. With further developments, iOCT systems may be essential for optimizing surgical outcomes by facilitating surgical procedures and intraoperative decision-making in corneal, cataract, and refractive surgery. [2].

## Data Availability

The data supporting this systemic review are from previously reported studies and datasets, which have been cited in this article.

## References

[1] Price F. W. (2021). Intraoperative optical coherence tomography: game-changing technology. *Cornea*.

[2] Ehlers J. P. (2016). Intraoperative optical coherence tomography: past, present, and future. *Eye*.

[3] Han S. B., Mehta J. S., Liu Y. C., Mohamed-Noriega K. (2016). Advances and clinical applications of anterior segment imaging techniques. *Journal of ophthalmology*.

[4] Meduri A., Bergandi L., Perroni P., Silvagno F., Aragona P. (2020). Oral l-cysteine supplementation enhances the long term-effect of topical basic fibroblast growth factor (bFGF) in reducing the corneal haze after photorefractive keratectomy in myopic patients. *Pharmaceuticals*.

[5] Muijzer M. B., Schellekens P., Beckers H. J. M., de Boer J. H., Imhof S. M., Wisse R. P. (2022). Clinical applications for intraoperative optical coherence tomography: a systematic review. *Eye*.

[6] Ehlers J. P., Tao Y. K., Farsiu S., Maldonado R., Izatt J. A., Toth C. A. (2013). Visualization of real-time intraoperative maneuvers with a microscope-mounted spectral domain optical coherence tomography system. *Retina*.

[7] Eguchi H., Hotta F., Kusaka S., Shimomura Y. (2020). Intraoperative optical coherence tomography imaging in corneal surgery: a literature review and proposal of novel applications. *Journal of ophthalmology*.

[8] Titiyal J. S., Kaur M., Nair S., Sharma N. (2021). Intraoperative optical coherence tomography in anterior segment surgery. *Survey of Ophthalmology*.

[9] Knecht P. B., Kaufmann C., Menke M. N., Watson S. L., Bosch M. M. (2010). Use of intraoperative fourier-domain anterior segment optical coherence tomography during descemet stripping endothelial keratoplasty. *American Journal of Ophthalmology*.

[10] Ehlers J. P., Dupps W. J., Kaiser P. K. (2014). The prospective intraoperative and perioperative ophthalmic ImagiNg with optical CoherEncE TomogRaphy (PIONEER) study: 2-year results. *American Journal of Ophthalmology*.

[11] Ray R., Barañano D. E., Fortun J. A. (2011). Intraoperative microscope-mounted spectral domain optical coherence tomography for evaluation of retinal anatomy during macular surgery. *Ophthalmology*.

[12] Ehlers J. P., Goshe J., Dupps W. J. (2015). Determination of feasibility and utility of microscope-integrated optical coherence tomography during ophthalmic surgery. *JAMA Ophthalmology*.

[13] Ehlers J. P., Kaiser P. K., Srivastava S. K. (2014). Intraoperative optical coherence tomography using the RESCAN 700: preliminary results from the DISCOVER study. *British Journal of Ophthalmology*.

[14] Ehlers J. P., Modi Y. S., Pecen P. E. (2018). The DISCOVER study 3-year results. *Ophthalmology*.

[15] Steven P., Le Blanc C., Velten K. (2013). Optimizing descemet membrane endothelial keratoplasty using intraoperative optical coherence tomography. *JAMA Ophthalmology*.

[16] Asif M. I., Bafna R. K., Sharma N. (2021). Microscope integrated optical coherence tomography guided descemet stripping automated endothelial keratoplasty in congenital hereditary endothelial dystrophy. *Clinical Ophthalmology*.

[17] Au J., Goshe J., Dupps W. J., Srivastava S. K., Ehlers J. P. (2015). Intraoperative optical coherence tomography for enhanced depth visualization in deep anterior lamellar keratoplasty from the PIONEER study. *Cornea*.

[18] Chen C. W., Lee Y. H., Gerber M. J. (2018). Intraocular robotic interventional surgical system (IRISS): semi-automated OCT-guided cataract removal. *The international journal of medical robotics + computer assisted surgery : MRCAS*.

[19] Cost B., Goshe J. M., Srivastava S., Ehlers J. P. (2015). Intraoperative optical coherence tomography-assisted descemet membrane endothelial keratoplasty in the DISCOVER study. *American Journal of Ophthalmology*.

[20] De Benito-Llopis L., Mehta J. S., Angunawela R. I., Ang M., Tan D. T. H. (2014). Intraoperative anterior segment optical coherence tomography: a novel assessment tool during deep anterior lamellar keratoplasty. *American Journal of Ophthalmology*.

[21] Fang W., Li Q., Fan J. (2020). Microscope-integrated intraoperative optical coherence tomography for anterior segment surgical maneuvers. *Translational Vision Science & Technology*.

[22] Fukumoto R., Inoue M., Ishida T., Koto T., Hirakata A. (2021). Adjustment of intraocular lens tilt during intrascleral fixation assisted by intraoperative OCT. *Journal of Cataract and Refractive Surgery*.

[23] Ghaffari R., Mortazavi M., Anvari P. (2018). Intraoperative optical coherence tomography to evaluate the effect of the eyelid speculum on corneal pachymetry during accelerated corneal cross-linking (9 mW/cm2). *Eye*.

[24] Juergens L., Michiels S., Borrelli M. (2021). Intraoperative OCT—real-world user evaluation in routine surgery. *Klinische Monatsblatter fur Augenheilkunde*.

[25] Lang S. J., Heinzelmann S., Böhringer D., Reinhard T., Maier P. (2020). Indications for intraoperative anterior segment optical coherence tomography in corneal surgery. *International Ophthalmology*.

[26] Liu Y.-C., Wittwer V. V., Yusoff N. Z. B. M. (2019). Intraoperative optical coherence tomography-guided femtosecond laser-assisted deep anterior lamellar keratoplasty. *Cornea*.

[27] Lytvynchuk L. M., Glittenberg C. G., Falkner-Radler C. I. (2016). Evaluation of intraocular lens position during phacoemulsification using intraoperative spectral-domain optical coherence tomography. *Journal of Cataract and Refractive Surgery*.

[28] Mazzotta C., Caragiuli S. (2014). Intraoperative corneal thickness measurement by optical coherence tomography in keratoconic patients undergoing corneal collagen cross-linking. *American Journal of Ophthalmology*.

[29] Muijzer M. B., Soeters N., Godefrooij D. A., van Luijk C. M., Wisse R. P. L. (2020). Intraoperative optical coherence tomography-assisted descemet membrane endothelial keratoplasty: toward more efficient, safer surgery. *Cornea*.

[30] Nair S., Kaur M., Titiyal J. S. (2021). Intraoperative optical coherence tomography guided imaging of incision-site descemet membrane dynamics during phacoemulsification. *JAMA Ophthalmology*.

[31] Scorcia V., Busin M., Lucisano A., Beltz J., Carta A., Scorcia G. (2013). Anterior segment optical coherence tomography-guided big-bubble technique. *Ophthalmology*.

[32] Shazly T. A., To L. K., Conner I. P., Espandar L. (2017). Intraoperative optical coherence tomography-assisted descemet stripping automated endothelial keratoplasty for anterior chamber fibrous ingrowth. *Cornea*.

[33] Siebelmann S., Händel A., Matthaei M., Bachmann B., Cursiefen C. (2019). Microscope-integrated optical coherence tomography-guided drainage of acute corneal hydrops in keratoconus combined with suturing and gas-aided reattachment of descemet membrane. *Cornea*.

[34] Siebelmann S., Matthaei M., Heindl L. M., Bachmann B. O., Cursiefen C. (2018). Intraoperative optical coherence tomography (MI-OCT) for the treatment of corneal dystrophies. *Klin Monbl Augenheilkd*.

[35] Steven P., Le Blanc C., Lankenau E. (2014). Optimising deep anterior lamellar keratoplasty (DALK) using intraoperative online optical coherence tomography (iOCT). *British Journal of Ophthalmology*.

[36] Titiyal J. S., Kaur M., Sahu S., Sharma N., Sinha R. (2017). Real-time assessment of intraoperative vaulting in implantable collamer lens and correlation with postoperative vaulting. *European Journal of Ophthalmology*.

[37] Urkude J., Titiyal J. S., Sharma N. (2017). Intraoperative optical coherence tomography-guided management of cap-lenticule adhesion during SMILE. *Journal of Refractive Surgery*.

[38] Saad A., Guilbert E., Grise-Dulac A., Sabatier P., Gatinel D. (2015). Intraoperative OCT-assisted DMEK. *Cornea*.

[39] Eguchi H., Kusaka S., Arimura-Koike E. (2017). Intraoperative optical coherence tomography (RESCAN 700) for detecting iris incarceration and iridocorneal adhesion during keratoplasty. *International Ophthalmology*.

[40] Siebelmann S., Steven P., Cursiefen C. (2016). [Intraoperative optical coherence tomography in deep anterior lamellar keratoplasty]. *Klin Monbl Augenheilkd*.

[41] Patel A. S., Goshe J. M., Srivastava S. K., Ehlers J. P. (2020). Intraoperative optical coherence tomography-assisted descemet membrane endothelial keratoplasty in the DISCOVER study: first 100 cases. *American Journal of Ophthalmology*.

[42] Kobayashi A., Yokogawa H., Mori N., Sugiyama K. (2016). Visualization of precut DSAEK and pre-stripped DMEK donor corneas by intraoperative optical coherence tomography using the RESCAN 700. *BMC Ophthalmology*.

[43] Juthani V. V., Goshe J. M., Srivastava S. K., Ehlers J. P. (2014). Association between transient interface fluid on intraoperative OCT and textural interface opacity after DSAEK surgery in the PIONEER study. *Cornea*.

[44] Hallahan K. M., Cost B., Goshe J. M., Dupps W. J., Srivastava S. K., Ehlers J. P. (2017). Intraoperative interface fluid dynamics and clinical outcomes for intraoperative optical coherence tomography-assisted descemet stripping automated endothelial keratoplasty from the PIONEER study. *American Journal of Ophthalmology*.

[45] Xu D., Dupps W. J., Srivastava S. K., Ehlers J. P. (2014). Automated volumetric analysis of interface fluid in descemet stripping automated endothelial keratoplasty using intraoperative optical coherence tomography. *Investigative Opthalmology & Visual Science*.

[46] Dai Y., Luo L., Liu Y. (2021). Intraoperative optical coherence tomography guided imaging of incision-site descemet membrane dynamics during phacoemulsification-reply. *JAMA Ophthalmology*.

[47] Tong C. M., Parker J. S., Dockery P. W., Birbal R. S., Melles G. R. J. (2019). Use of intraoperative anterior segment optical coherence tomography for bowman layer transplantation. *Acta Ophthalmologica*.

[48] Siebelmann S., Horstmann J., Scholz P. (2018). Intraoperative changes in corneal structure during excimer laser phototherapeutic keratectomy (PTK) assessed by intraoperative optical coherence tomography. *Graefe’s Archive for Clinical and Experimental Ophthalmology*.

[49] Schmidt E. M., Stiefel H. C., Houghton D. C., Chamberlain W. D. (2019). Intraoperative optical coherence tomography to guide corneal biopsy: a case report. *Cornea*.

[50] Ruland K., Bouldin T. W., Davis R. M., Fleischman D. (2018). Intraoperative optical coherence tomography-assisted retrocorneal fibrous membrane biopsy and excision. *American Journal of Ophthalmology Case Reports*.

[51] Sharma N., Urkude J., Chaniyara M., Titiyal J. S. (2017). Microscope-integrated intraoperative optical coherence tomography-guided small-incision lenticule extraction: new surgical technique. *Journal of Cataract and Refractive Surgery*.

[52] Nagpal R., Shakkarwal C., Agarwal R., Bafna R. K., Maharana P. K., Sharma N. (2021). Quantitative analysis of gap between the intraocular lens and posterior capsule using microscope-integrated optical coherence tomography in eyes undergoing phacoemulsification. *Clinical Ophthalmology*.

[53] Hirnschall N., Norrby S., Weber M., Maedel S., Amir-Asgari S., Findl O. (2015). Using continuous intraoperative optical coherence tomography measurements of the aphakic eye for intraocular lens power calculation. *British Journal of Ophthalmology*.

[54] Meduri A., Oliverio G., Severo A. A., Camellin U., Rechichi M., Aragona P. (2022). Double safe suture during cataract surgery on post radial keratotomy patients using clear corneal incisions. *European Journal of Ophthalmology*.

[55] Meduri A., Urso M., Signorino G. A., Rechichi M., Mazzotta C., Kaufman S. (2017). Cataract surgery on post radial keratotomy patients. *International Journal of Ophthalmology*.

[56] Sachdev M. S., Malik R., Gupta H., Sachdev R., Sachdev G. S. (2020). Femtosecond laser-integrated anterior segment optical coherence tomography to detect preexisting posterior capsular dehiscence and increase safety in posterior polar cataracts. *Journal of Cataract and Refractive Surgery*.

[57] Chen W., Lin Z., Zhu Q. (2021). Intraoperative optical coherence tomography for the assessment of posterior capsular integrity in pediatric cataract surgery. *Journal of Cataract & Refractive Surgery*.

[58] Titiyal J. S., Kaur M., Shaikh F., Goel S., Bageshwar L. M. S. (2020). Real-time intraoperative dynamics of white cataract-intraoperative optical coherence tomography-guided classification and management. *Journal of Cataract and Refractive Surgery*.

[59] Chen C.-W., Francone A. A., Gerber M. J. (2019). Semiautomated optical coherence tomography-guided robotic surgery for porcine lens removal. *Journal of Cataract and Refractive Surgery*.

[60] Ehlers J. P., Tao Y. K., Farsiu S., Maldonado R., Izatt J. A., Toth C. A. (2011). Integration of a spectral domain optical coherence tomography system into a surgical microscope for intraoperative imaging. *Investigative Opthalmology & Visual Science*.

[61] Ehlers J. P., Srivastava S. K., Feiler D., Noonan A. I., Rollins A. M., Tao Y. K. (2014). Integrative advances for OCT-guided ophthalmic surgery and intraoperative OCT: microscope integration, surgical instrumentation, and heads-up display surgeon feedback. *PLoS One*.

[62] El-Haddad M. T., Tao Y. K. (2015). Automated stereo vision instrument tracking for intraoperative OCT guided anterior segment ophthalmic surgical maneuvers. *Biomedical Optics Express*.

[63] Han S. B., Liu Y. C., Mohamed-Noriega K., Mehta J. S. (2021). Advances in imaging technology of anterior segment of the eye. *Journal of Ophthalmology*.

